# Priorities for science to overcome hurdles thwarting the full promise of the ‘digital agriculture’ revolution

**DOI:** 10.1002/jsfa.9346

**Published:** 2018-10-22

**Authors:** Mark Shepherd, James A Turner, Bruce Small, David Wheeler

**Affiliations:** ^1^ Farm Systems and Environment Group, AgResearch Ltd Ruakura Research Centre Hamilton New Zealand

**Keywords:** technology, precision agriculture, digital agriculture, digital science, digitalisation, value chain

## Abstract

Abstract

The world needs to produce more food, more sustainably, on a planet with scarce resources and under changing climate. The advancement of technologies, computing power and analytics offers the possibility that ‘digitalisation of agriculture’ can provide new solutions to these complex challenges. The role of science is to evidence and support the design and use of digital technologies to realise these beneficial outcomes and avoid unintended consequences. This requires consideration of data governance design to enable the benefits of digital agriculture to be shared equitably and how digital agriculture could change agricultural business models; that is, farm structures, the value chain and stakeholder roles, networks and power relations, and governance. We argue that this requires transdisciplinary research (at pace), including explicit consideration of the aforementioned socio‐ethical issues, data governance and business models, alongside addressing technical issues, as we now have to simultaneously deal with multiple interacting outcomes in complex technical, social, economic and governance systems. The exciting prospect is that digitalisation of science can enable this new, and more effective, way of working. The question then becomes: how can we effectively accelerate this shift to a new way of working in agricultural science? As well as identifying key research areas, we suggest organisational changes will be required: new research business models, agile project management; new skills and capabilities; and collaborations with new partners to develop ‘technology ecosystems’. © 2018 The Authors. © 2018 The Authors. *Journal of The Science of Food and Agriculture* published by John Wiley & Sons Ltd on behalf of Society of Chemical Industry.

## INTRODUCTION

Innovation has always been present in agriculture: how else could we have fed a world population that has grown fourfold in the last 100 years to upwards of 7 billion? This innovation needs to continue if we are to sustainably supply safe and nutritious food to a population that will grow to over 9 billion by 2050 and on a planet where resources are becoming scarcer. All the signs are that our increasingly powerful digital technologies have great potential to drive the ‘Fourth Industrial Revolution’.^1^ This will include technological solutions for meeting these food supply challenges: ‘the Third Green Revolution’.^2^

Digital technology is not new to agriculture. One could argue that its beginnings are rooted in the late 1980s^1^ through the concept of ‘precision agriculture’ (PA) with the use of global positioning system (GPS) guidance on tractors^3^ and yield mapping.^4^ PA agriculture has since developed to use a range of proximal and remote‐sensing methods to monitor within‐field variations in soil and crop attributes and linking these to variable‐rate technologies (VRTs) to guide inputs of, for example,. water and nutrients.^5^ The next challenge for PA is to harness the potential of the collected data to provide bespoke decisions.^6^ This is becoming increasingly technically possible now that ‘computers are rapidly encroaching into areas that used to be the domain of people only, like complex communication and advanced pattern recognition’.^7^ And, as we explain in this paper, it is this growing ability to utilise the technology to convert precise data along the value chain into actionable knowledge to drive and support complex decision‐making on‐farm and along the value chain that will distinguish the digitaliation of agriculture (‘digital agriculture’) from PA; enabling the move ‘from precision to decision’.

There is a general assertion that digital technologies have potential to truly transform agriculture. There are numerous examples of both the agricultural industry and governments preparing for a technology‐driven future, based on this assertion. For example, the European Union is exploring how its Common Agricultural Policy will encompass digital agriculture in the future.^8^ Similarly, technology is reported to be transforming the lives of India's farmers.^9^ However, for the potential to be truly realised, the technology has to be implemented on a large scale. History suggests this could be a slow process. For example, Dutch studies have shown that about 20% of dairy farmers have on‐cow sensors for oestrus detection but other sensors are hardly adopted yet.^10^ And even though VRT is widely available, a USDA Economic Research Report showed that only about 20% of farmers in the USA have adopted the technology.^11^ Autosteer (GPS guidance in tractors) took 22 years to be taken up by 77% of Australian grain growers.^12^ Despite numerous authors confirming the economic and environmental benefits of PA there is still a low rate of adoption reported by academic surveys and professional reports (see citations in Pierpaoli *et al*.^13^).

The aim of this paper, therefore, is to consider how science can support uptake of these technologies. We believe this is important for two reasons: the huge food production challenges that we face will require new solutions and approaches that digital technology promises; but, as we discuss in this paper, there needs to be strong, science‐based, evidence that the technologies are delivering sustainable food production systems, with the benefits of these technologies shared among agri‐food sector stakeholders in a way that gives farmers, consumers and regulators confidence in the outcomes.

Digital technologies have the potential to impact on agriculture, horticulture and animal production units. Furthermore, it is expected that the entire food chain will benefit because of the integrated flow of information both upstream and downstream.^8^ However, for brevity we have focused this paper predominantly on on‐farm cropping and animal production systems.

### The future of food production?

Predicting the future is fraught with difficulties. However, there is a strong consensus that future food production will be shaped by several megatrends that challenge the status quo. These were signalled as far back as 2001 by Kohl,^14^ and recently have been further elucidated by Hajkowicz and Eady^15^ as a hungrier world, a bumpier ride, a wealthier world, choosier customers, and transformative technologies.

The implication of these trends is that agribusiness will come under increasing scrutiny by communities, regulators and consumers.^16^ This will be driven by increasing environmental pressures from climate change and a world that is reaching its ecological limits, combined with growing middle‐ and high‐income classes who place greater requirements on ethically produced and healthy food, and who are empowered by access to real‐time information regarding the provenance and production of what they eat. Access to synthetic foods that can meet the requirements of these middle‐ and high‐income classes will also increase competition with food produced using traditional agricultural practises.

## DEFINING ‘DIGITAL AGRICULTURE’

We define digital agriculture as the use of detailed digital information to guide decisions along the agricultural value chain. This can include the use of high‐volume, variable source data (‘big data’), to produce actionable knowledge. Importantly, it is not restricted to the farm or animal production unit but can span all or part of the value chain; indeed, one of the transformational aspects of a digitised world is the potential for an almost direct link between consumer and food producer. Wolfert *et al*.^17^ call this ‘smart farming’: ‘a development that emphasizes the use of information and communication technology in the cyber‐physical farm management cycle’. Smart farming is, therefore, more than PA as it links farmer, consumer and other value‐chain stakeholder information. This means consumers can make purchases based on information about the farm producing the food, and farmers can make production decisions based on information about consumer purchases.

This concept of digitalisation of agriculture fits well with the standard definition of business digitalisation: ‘digitalisation most often refers to enabling, improving and/or transforming business operations and/or business functions and/or business models/processes and/or activities, by leveraging digital technologies and a broader use and context of digitised data, turned into actionable, knowledge, with a specific benefit in mind’.^18^

### The technology driving the digital agriculture revolution

Digital agriculture's change will largely be in the form of new technologies, including improved sensor capability, improved data connectivity, and computer‐based artificial or augmented intelligence (AI) decision support and self‐learning systems. The potential for transformation arises from the co‐evolution, convergence, and integration of a wide range of these existing, emerging, and future technologies.

#### *Sensors*

Sensors are the technological units that are likely to have the most impact with regard to facilitating future cost‐effective data collection. The rate of sensor development is increasing, while their unit price is decreasing. The increasing miniaturisation of sensors and the ability to fit more transistors on a single chip will lead to sensors with multitasking capability in the very near future. It has been argued that miniaturisation was the main driver of the ‘Third Industrial Revolution’,^19^ which, in itself, set the foundation for the ‘Fourth Industrial Revolution’.^1^ Cost and miniaturisation combined mean that affordable sensors have the potential to soon become common within the farming environment. This is evidenced in other areas by the huge consumer uptake of compact, portable, smarter devices that work faster and have multiple added features.^20^ Challenges still exist, however, such as their ability to function in rugged conditions (e.g. attached to animals in a herd environment). Also, meeting power requirements becomes a challenge for portable devices such as highly accurate high‐frequency GPS sensors required to operate in situations where battery changes cannot occur.

Examples already in use include electronic identification of livestock. Furthermore, this information can be used to link with individual milk meters and can also trigger individual rations. In cropping systems, sensors on combine harvesters have long been used to generate yield maps when linked to GPS. Such data, often from multiple sources, that can be captured, analysed, and used for decision‐making, is pivotal to realising the full potential from digital agriculture. The availability of low‐cost sensors lowers the cost of assembling these big data sets. The next challenge is managing the data.^6^ We can think about this in terms of capacity to manage, move, and store data, as well as analytical capability.

#### *Telecommunication and data storage*

The cost of both data transfer and storage is rapidly declining, making data‐based technology even more affordable for farmers. The cost of wireless data transfer has dropped 75% in the past 4 years, while the cost of data storage has dropped by 97% per gigabyte of storage in the last 10 years. Data storage considerations include the place of storage (legal jurisdiction); privacy and accessibility; backing up and data security; the division between cloud‐based and on‐site storage; and the ‘volatility’ of data (how long data needs to be kept), which is still evolving, partly as the amount of data that is required to be transferred increases.

#### *Analytics*

Analytics is the capability available to analyse data, and it is rapidly increasing. In the past 10 years, there has been a 32× increase in computing power for the same cost, with the current rate of change in analytical ability expected to increase exponentially in the future. This will increase the ability to analyse big data more rapidly, potentially even allowing for real‐time analysis. It will also increase our ability to use AI to deliver solutions. Thus, analytics has potential to significantly impact on the way agriculture operates within society, moving us from ‘hindsight’ to ‘foresight’.

Most agricultural systems currently use the analytical techniques associated with descriptive and diagnostic analytics. That is, they establish ‘what happened’ and ‘why it happened’ and as such are hindsight focused. Digitising agriculture will take these systems to predictive (what will happen) and prescriptive (how can we make it happen) analytics, which are future focused. In the case of predictive analytics, the unknown event of interest is often in the future. However, predictive analytics can be applied to any type of unknown, whether it be past, present, or future. Specifically, it is an area of statistics that deals with extracting information from data, combining it with rules, algorithms, and occasionally external data, and using it to predict trends and behaviour patterns, and the likelihood of a situation occurring. The question then becomes: Will this provide the potential for producers and others in the value chain to more effectively meet increasing regulatory compliance and scrutiny from consumers? The fact that predicting the future is fraught implies that digital agriculture needs to provide flexibility so that the emergent trends can be readily and quickly addressed. Prescriptive analytics, based on wide range of data collected from multiple sensors, increases opportunities for this to occur.

#### *Other technologies*

Connolly^21^ listed eight digital technologies that are predicted to transform agriculture. That list includes what has already been mentioned, plus drones, robots, and blockchain. Drones are possibly the first technology that comes to mind when digital agriculture is mentioned. However, we consider them as a vehicle for transporting sensors, able to collect information (often visual) to aid decision‐making. Increasingly, there is a thought that they could also administer treatments (e.g. laser treatment or spot chemical treatment of weeds). Therefore, they could also be considered as robots. Robots are already working on farms, saving on labour and removing some of the mundane tasks (e.g. robotic milking). Amongst other benefits, this technology has removed the drudgery of very early morning manual milking for farmers, and there is anecdotal evidence that this makes dairy farming a more attractive proposition for new entrants (Andersen S, personal communications). Blockchain ‘is an incorruptible electronic ledger that can track each transaction of a food item's journey through the food chain’^21^ and therefore will allow tracking of food along the value chain, making proof of provenance easier for the consumer.

#### *Connectivity*

Access to reliable fast broadband, for connection to the Internet of Things and ability to rapidly transfer large amounts of data, is essential for rural communities to participate in digital agriculture. Yet, rural areas are among those most excluded from fast broadband developments.^22^ Policymakers and farmer organisations have acknowledged the ‘digital divide’ between rural and urban areas, and there is evidence that governments recognise addressing this is a priority.^8^ However, a number of private‐sector projects are under way to make ultrafast, universal broadband a reality. Companies currently involved in developing universally available broadband include Oneweb, SpaceX, Samsung, Google, and Facebook. The development of long‐range‐low‐energy connectivity technologies (such as BLE, NB‐IoT, LorA, SigFox) that are relatively cheap solutions for rural areas and agricultural activities are another option.^23^ Nevertheless, it will take time to address connectivity issues, and this is perhaps the single largest technical challenge that will limit uptake of digital agriculture.

## HOW DIGITAL AGRICULTURE CAN HELP DELIVER THE FUTURE: NEW ZEALAND AS AN EXAMPLE

Farming is New Zealand's largest industry, and New Zealand is a major exporter of milk and red meat products. Recent analyses of the industry indicate that the megatrends – a hungrier world, a bumpier ride, a wealthier world, choosier customers, and transformative technologies – will increase the regulatory requirements and compliance costs placed on agribusiness from inputs to the farm to the supply chain, while continuing to drive commodity prices downward.^24^, ^25^

**Figure 1:**
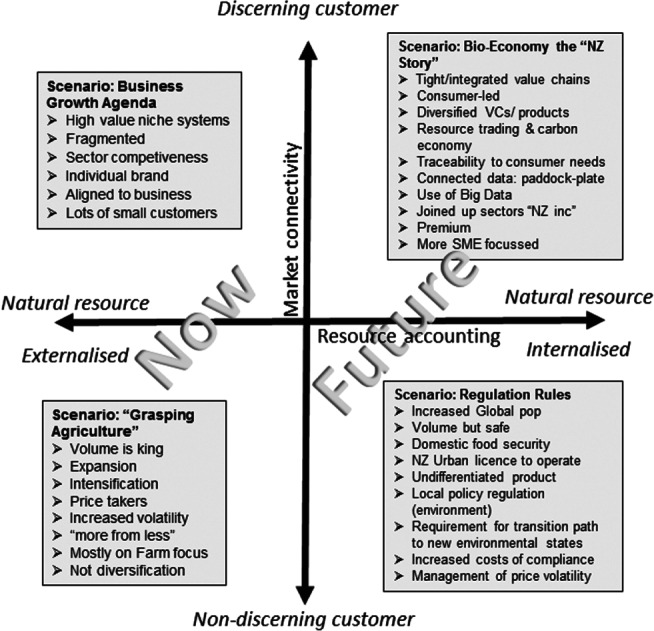
Summary of four potential agricultural scenarios, as identified by Saunders *et al*.^26^

Saunders *et al*.^25^ identified four possible scenarios for New Zealand agriculture depending on how compliance costs were accounted for and the target consumer (Fig. 1). Shadbolt *et al*.^24^ suggested two strategies that would enable the New Zealand dairy industry to continue to survive under increasing community, regulatory, and consumer scrutiny: either pursue economies of scale and efficiency to reduce commodity production costs, or realise added value by creating premium products targeted at differentiated, niche markets. From the compiled evidence, we can assume that, in the future: environmental standards and regulations will become more stringent; origin and traceability will become critical to international market access for agricultural products; opportunities to add value to products through demonstration of provenance and traceability of product or management systems will increase; and decision‐making on‐farm will be increasingly data based and robust. In other words, consumer demands are likely to push much of the industry to the right‐hand side of Fig. 1.

These future scenarios provide hugely complex challenges for agriculture. However, the increasing ease with which digital technologies will allow the collection, management, and transfer of information – and its use to support decision making – offers real opportunities to meet the requirements demanded by these future scenarios, as illustrated in Table 1.

**Table 1:** Science and technology requirements required to support possible future agricultural scenarios, adapted from Saunders *et al*.^26^

Overall goal	Technologies	Science challenges
To demonstrate value‐add attributes along the supply chain to consumers		Evidence for the consumers that the use of practices, sensors, traceability, and analytics achieves the desired attributes
	Sensors, analytics and traceability	
New supply chain business models		Can information from consumers feed back along the value chain to improve production and processing methods to achieve desired food attributes?
Improve productivity	Sensors and analytics	Can the tools deliver lower cost productivity without unforeseen consequences? Is it practically possible to manage (e.g. irrigate, fertilise, spray) at the sub‐field scale indicated by the sensors/analytics?
To lower the cost of achieving compliance	Sensors, analytics and decision support systems	Can the tools deliver improved decision‐making to achieve compliance with environmental, health, safety, and animal welfare requirements at lower cost than current approaches?
To lower the cost of demonstrating compliance to regulators	Sensors, analytics and decision support systems	Evidence that adoption of practices, sensors, and analytics achieves the desired goals relating to environmental, health, safety, and animal welfare requirements

In summary, the technologies must be able to help farmers to grow viable agribusinesses by leveraging the increasingly discerning demands of consumers and regulators. While this will be achieved by integrating the technologies into the farm system to aid better decision‐making, other aspects could include saving labour (e.g. robotic milking, driverless tractors) and automating administrative systems, such as ordering materials, managing health and safety, and providing audit data for regulatory compliance and product provenance.

Table 1 highlights the need for technological support to demonstrate compliance to regulators and/or customers. This might be information as diverse as evidence of desired farm practice (e.g. recording placement and rate of fertiliser, evidence of stock exclusion from waterways), monitoring aspects of environmental footprint (e.g. drain/stream nutrient contents), or calculating a footprint from a range of input data monitored in real time on the farm. All of these would alleviate a reporting burden currently placed on the farmer and serve as evidence of provenance for the consumer and regulator. Importantly, and a critical advantage that digital agriculture could deliver, is that the same information can be packaged to support decision‐making, demonstrate compliance to regulators and/or processors and provenance to consumers.

The availability of technology will not be a driver for uptake in itself. Adoption must be supported by evidence that digital agriculture can provide benefits desired by farmers, processors, regulators and consumers, while also enhancing farming identities. The potential social benefits of digitilisation are many, including increased production for less inputs, less environmental damage and products with better environmental credentials, better working conditions for employees, more efficient transportation and logistics, better temporal delivery of products for consumer needs, and improved ability to meet changing consumer desires and expectations regarding provenance and proof of socio‐ethical factors, and responsible, sustainable production.

The key question for agricultural scientists then becomes: How can science ensure stakeholders have the confidence that adoption of digital agriculture will deliver these benefits they seek, while ensuring transparency and equitable sharing of benefits from access to and use of digital technologies and data?

### Barriers to uptake of technologies

Despite the potential that technology offers, past experience suggests that adoption could be slow. Recent research suggests that the critical mass required to create a social tipping point, whereby a minority group, such as adopters of PA, can initiate a cascading change of social behaviour, is about 25% of the relevant population.^27^ Despite decades of development, as the earlier example cited of VRT demonstrated, uptake has been slow. There are other cases; for example, the history of crop modelling. A recent review concludes that even after over 40 years of development there is little evidence this activity has produced many on‐farm benefits.^28^ In another, Corti *et al*.^26^ conclude that over the past 25 years there has not been much useful progress in remote sensing for corn. These cases illustrate how difficult it will likely be to fulfil the promise of digital agriculture. The challenge is clear: avoid a future where digital technologies are embedded in the science community but have not fulfilled expectations in sustainable food production. While there will remain challenges with technologies themselves (e.g. rural connectivity), there is plenty of evidence to suggest that the main barriers to uptake will be socio‐ethical.

#### *Data ownership, use and trust*

Wolfert *et al*.^17^ postulate that big data are central to the transformational change offered by digital agriculture. However, lack of trust is a current barrier to uptake of digital agriculture in addition to barriers experienced by PA, because of storage and analysis of detailed farm‐specific data to inform decision‐making. Farmers identify concerns regarding transparency (who will have access to data and how they will make use of it?)^29^ and equity (who benefits from access to and use of farmers' data?).^17^, ^30^ Exacerbating the lack of trust is a sense that political and legal control of big data is lagging behind technical developments,^31^ with the perceived risk that control of data will reside with technology providers, rather than farmers as technology users. This raises questions regarding data governance, while simultaneously enabling integration of data to provide actionable knowledge.^17^

#### *Competing business models*

It has been postulated that big data will drive the development of new business models to leverage value from the data.^17^ Here, tensions can possibly arise; for example, between commercial companies looking to monetise big data and public institutions that publish open data. More direct linking of farmers and consumers through a value chain also challenges some of the current business models for agricultural product delivery, but also offers opportunities for new models. How these tensions play out, combined with the aforementioned questions around data ownership, transparency, and trust, will determine how business models evolve to service the food production chain.^29^ The two extremes, as postulated by Wolfert *et al*.,^17^ are closed systems with the farmer integrated as part of the value chain *versus* open, highly collaborative systems that give all stakeholders in the value chain flexibility in the way they operate.

#### *Unclear benefits*

The farmer‐perceived barriers to uptake of digital agriculture are common to the adoption of other agricultural technologies, including PA:^13^ high investment costs, complexity of use, lack of fit with the particular farming context, and unclear benefits relative to current practices.^32^ For example, all of these reasons were cited as to why Dutch dairy farmers tend to delay investments in sensors.^33^ This suggests that uptake of digital agriculture could follow a similar pattern to that of PA, which tends to be taken up by more educated farmers familiar with PA, confident in the use of information and communications technology and using farm consultants, with a larger farm (>500 ha) with good soil quality, and aiming to implement more productive agricultural practice to respond to growing competitive pressures.^13^

#### *Changing farmer roles, practices, and identities*

Digital agriculture can become an enabler of different forms of ‘good farming’, and thus how farmers perceive the technologies fitting with their own farming identity.^29^, ^34^ Providers and farmers utilising these technologies refer to them as means for agriculture to increase efficiency, effectiveness, and productivity, including more efficient farm management.^34^, ^35^ ‘Good farmers’ are those who follow the data, not their gut.^34^, ^35^ In this context, digital agriculture becomes an enabler of further industrialisation of agriculture (intensification, increasing farm size, and use of technological and chemical inputs).^29^, ^34^ This is potentially a more likely future based on the current need for capital to invest in the technologies, so the technologies are more likely to be adopted by larger commercial farms. There is also a perceived risk of increasing reliance on technical experts and the technology resulting in a loss of tacit knowledge and that farmers may become ever more reliant on the technology for decision‐making.^31^ This is the closed systems,^17^ big farming,^29^ or regulation rules^25^ scenario (Fig. 1).

Another, though less common, framing of digital agriculture is around its role in solving societal concerns regarding industrialisation of agriculture.^35^ For example, by enabling increased transparency of food provenance, animal welfare and environmental, and facilitating easier sharing of information between farmers and consumers and provision of products from farmers directly to consumers.^17^ This is the open, highly collaborative systems,^17^ big data for everyone,^29^ and bio‐economy New Zealand story.^25^

## HOW CAN SCIENCE HELP REALISE THE DESIRED BENEFITS FROM DIGITAL AGRICULTURE?

There is an expectation that digital technologies will help to solve the challenge of sustainable food production, providing new solutions to old and new problems. Saunders *et al*.^25^ identify two research questions that will support adoption of digital agriculture (Table 1): Do the technologies (sensors, analytics, and decision support systems) work in a farm system (and along the value chain)? And, what is the scientific evidence to give consumers and regulators confidence that the technologies are delivering the expected benefits (e.g. environmental compliance, animal welfare, or food provenance)? To these two questions we add two more in light of the socio‐ethical issues identified earlier: How are the different benefits of adoption shared among technology providers, farmers, regulators and value chain stakeholders?^27^ And, what are the potential implications for farmer access to technologies, for their practice and identity?^35^

Thus, while it is easy to be seduced by the technology, the role of science is to evidence and support the design and use of digital technologies to realise these beneficial outcomes and avoid unintended consequences. This requires consideration of data governance design to enable benefits of digital agriculture to be shared equitably^17^, ^35^ and how digital agriculture could change agricultural business models; that is, farm structures, the value chain and stakeholder roles, networks and power relations, and governance,^35^ as happened with wider adoption of tractors and the introduction of pesticides in the 1950s.^17^

We argue that this requires transdisciplinary research at pace, including explicit consideration of the earlier socio‐ethical issues, data governance and business models,^35^, ^36^ alongside addressing technical issues, as we now have to simultaneously deal with multiple interacting outcomes in complex technical, social, economic. and governance systems and work with new disciplines that we have not historically worked with. The exciting prospect is that digitalisation of science can enable this new, and more effective, way of working. The question then becomes: How can we effectively accelerate this shift to a new way of working in agricultural science? Our suggestions are in the following.

### Address key socio‐ethical priorities

As Fleming *et al*.^29^ have previously noted, the key barriers to be addressed for digital technologies to deliver beneficial outcomes tend to be socio‐ethical. To address issues of data ownership, transparency, and trust it is critical to include farmers (and other relevant stakeholders) in design of technologies, as well as in the design of new governance and business models that will enable desired benefits from digital agriculture.^30^, ^35^, ^36^ This increases transparency in the innovation process, enhancing stakeholder trust in technologies,^30^ ensures fit‐for‐purpose solutions, consideration of wider implications for farmer roles, practices and identities in agriculture,^36^ and implications for the sharing of benefits among stakeholders. For example, what business models simultaneously provide privacy, transparency, and desired benefits for farmers, as well as a sustainable revenue stream for technology providers?^17^, ^35^

### Address key technical priorities

#### *Demonstrate the distribution of different benefits from adopting an integrated packaged of digital technologies*

While numerous authors confirm the economic and environmental benefits of PA,^13^ the lack of a value proposition has been cited by farmers as one of the barriers to embracing digital agriculture.^32^ Examples of value assessment are starting to appear, however. For example, a Dutch study demonstrated a favourable financial benefit from investment in cow oestrus detection.^37^ Interestingly, the same conclusion was reached in another Dutch study, demonstrating that investment now in automated oestrus detection, as an example of a ‘mature’ technology, was financially sound but that delaying investment in an example of an emerging technology, where there was still room for further technical improvement, was the financially sound decision.^10^ This points to the need to factor in the maturity of the technology when considering its value.

Furthermore, what is potentially missing are examples of different benefits desired by farmers through digital agriculture, particularly nonfinancial. For example, robotic milking can dramatically improve a farmer's lifestyle by removing the drudgery of early‐morning milking, as described earlier. Finally, the benefit of digital agriculture is more likely to accrue when data collection, value chain, and creation of actionable knowledge are coordinated. The value of using digital technologies is greater when these technologies are integrated effectively, rather than as individual incremental component changes.

This points to the need for wider analysis of the costs and benefits delivered, including adoption as individual and an integrated package of technologies. There have been discussions in Europe to develop a methodology to support cost–benefit analysis of PA,^38^ and we would argue that this is also a priority for digital agriculture, along with including consideration of wider benefits and costs relevant to different stakeholders. This assessment of value needs to be built into a project at the outset, and to consider the wider impacts on the farmer's lifestyle (e.g. time spent on different farm management tasks, roles of the family on the farm, health and safety, and farm succession) of integrated packages of digital technologies.

#### *More examples of ‘actionable knowledge’*

Another priority is demonstrating clear practical cases of ‘actionable knowledge’ derived from sensors and analytics, so that the management cycle ‘sense–analyse–act’ can be closed. For example, one might be able to measure variability at a resolution of a square metre and be able to analyse this at this resolution, but does a farmer have the equipment to control this process at the same resolution? This is a potential barrier to uptake, if the equipment (e.g. irrigator, harvester, or sprayer) is relatively much more expensive than sensors. Understanding these practical implications around ‘size of management unit’ is central to providing credible solutions. However, it could also drive innovation; for example, building equipment that allows management at a smaller scale if there was a clearly defined benefit.

#### *Provide supporting validation*

Providing supporting evidence that a component technology is doing what it promises is relatively straightforward. For example, accuracy limits for GPS units are provided by manufacturers and can be tested by the user. Ground‐truthing is another method. Or making observations or chemical analysis to compare with predictions made by sensors. The challenge of validation, however, can become more difficult around computer‐aided decisions and proving that, in this case, a digitally derived option is performing better than alternative choices.

#### *Develop more examples of (big) data and computer‐aided decision support*

Agricultural researchers will play a pivotal role in this context in developing and defining the decision support systems required for whole farm management that enables optimal returns on inputs while also preserving resources and the environment, and meeting consumers' needs. There is still a long way to go before computer‐aided or computer‐made decisions become routine, but the promise these systems hold is great. At present, there is still a heavy reliance on the user to interpret the data to make decisions. For example, a review of 139 sensor systems to support animal health management decisions on dairy farms in the Netherlands found no examples of integration with other information to produce advice.^39^ Furthermore, all computer‐made decision systems that are currently being developed have a heavy reliance on past science. However, AI‐supported decision‐making could provide agricultural innovators with much greater confidence, thereby fuelling a surge in the development of value‐added products that serve well‐defined and tested niche markets that have, at least theoretically (via forecasting), been proven to be successful before they are even released.

#### *Develop integrated solutions along the value chain*

Digitalisation throughout the whole of the value chain may produce extra value throughout the chain. There is sufficient technology now available to link the customer through the value chain to the farmer. Such technology has the potential to be disruptive to current supply chains; for example, shorter supply chains through online sales and more prescriptive farming.^40^ The missing knowledge is how the farmer adapts management practices and product attributes to link with customer expectations. This might range from enhancement indices of sustainability through to farms modifying their animal breeding and selection methods to provide particular products, or altering grazing management to enhance product characteristics. Here, advanced analytics are likely to play a key role in predicting the parameters in which the greatest value lies. However, a further challenge is that the digitalisation may result in new business models that are quite different to the current farm business models;^39^ this will also influence the questions that we are asked to address as scientists.

Similarly, business models along the whole value chain will need to change. The success of digital agriculture to provide integrated solutions along the value chain will be dependent upon data governance and business models that ensure privacy and security, while also simultaneously enabling integration of data to provide actionable knowledge, and equitable sharing of benefits among the different stakeholders in the chain. This is needed to support the sharing of data and information in return for an increase in value to all participants. This will be challenging for value chains that have traditionally withheld strategic information from other parts of the chain. Here, digital technologies could be used to gain a further competitive advantage and capture the maximum value for their own returns. This is a within‐value‐chain competitive model. Digital agriculture, alternatively, under different data governance and business models, has potential to enable collaboration and trust within value chains.^17^, ^29^, ^34^ Here, the science questions may concern the use of technologies such as blockchain and social science on governance, trust, and collaboration.

### Change the way we work

Table 2 summarises the main priority areas that we believe need to be addressed if agricultural science organisations are to support the development and implementation of digital technologies to bring about more sustainable food production systems.

**Table 2:** Recommendation for priority areas for scientists/institutions to enable the potential benefit of digitalisation of science to be captured

	Priority	Issue to be resolved
Digitalisation of science	Move from reductionist approaches	The emerging science of big data, data veracity, and analytics will require a very different skillset.
Interdisciplinary research	Move out of silos	Solutions are beyond the scope of a single discipline or area of research practice, requiring interdisciplinary and transdisciplinary approaches. Digitalisation can serve as a catalyst.
Organisational change	Reassess business models	‘New organizational structures, processes and business models that leverage ever‐advancing technology and human skills’.^7^ The scientific value of big data will often derive from combining data from multiple sources; the current research business models for accessing and using such data may need to change to enable transparent data sharing.
	Pace of change	The predicted pace of technology progress will also provide challenges for the traditional 3–5 year funding cycles, the annual funding calls, and the time that it traditionally takes to achieve scientific progress.
	Beyond business as usual	Many organisations cannot visualise the paradigm shift that digital agriculture could cause, focusing more on the here and now; that is, at the tactical and operational levels.^41^
	New skills, capabilities	Skills and technological capability to operate in this field; will need to be able to apply scientific knowledge to capture, interpret, share, and apply digital information appropriately.
	New partnerships	One way for research organisations to develop new skills and capabilities is through partnerships or collaborations among partners that have not traditionally worked together; for example, linking agricultural scientists with technology companies (‘technology ecosystems’).
	Project management	The funding and project management required to optimise outcomes from digital‐based programmes has the potential to be different to conventional funding methodologies based on hypothesis‐driven reductionist science carried out in commercial or public‐funded research organisations.

#### *Digitalisation of science*

Digital technology has the potential to change the way we are able to do science. This will be a challenge, but it can potentially reap large rewards in terms of speed of progress. The challenge then becomes: How do we, as scientists, leverage digitalisation to accelerate the pace of our science given the pressing need to address the current challenges agriculture faces and the pace of technological progress?

While traditional roles of data reduction and analysis will still have their place, the emerging science of big data, data veracity, and analytics will require a very different skill set. Digitalisation will challenge the way we understand the problem, undertake or interpret data collected, and provide solutions. Deep learning will challenge the way we think about data and the role scientists have in understanding that data. The focus shifts from not necessarily fully understanding the data, but rather understanding the relationships between different data and processes.

Digitalisation challenges some of the fundamental ways we undertake science. Variability in the data is sought after and useful; fostering a move away from historical reductionism, where a problem is reduced to a single testable hypothesis. Rather, an understanding of the relationships between data is used not only to solve the problem, but also to identify additional or hidden issues. These relationships are likely to be better identified by AI than by humans. However, an understanding of human factors (in the case of potential training biases in AI), how aspirations and cultural values are incorporated, and human interpretation of difficult data points in data veracity (that may be considered either invalid or instead be valid points at the extreme edge of the acceptable range) is going to become increasingly important with any automatic systems tending towards less and less human intervention.

Digitalisation could also challenge the way we analyse literature and undertake experiments. The thinking is already that the sheer volume of knowledge produced is now impossible for scientists in a domain to keep up with, leading to a narrower disciplinary focus, which in turn risks missing potential solutions outside a discipline. It has been suggested that ‘literature‐related discovery’ has potential for the identification of unexplored hypotheses by linking previously unconnected knowledge domains,^42^ especially if AI could be integrated into the scientific process.^43^

#### *Interdisciplinary and transdisciplinary research*

Interdisciplinarity is considered a more effective method to address complex problems ‘whose solutions are beyond the scope of a single discipline or area of research practice’;^44^ the field of environmental science is an example.^45^ The challenges for agriculture require more integrated solutions; and digitalisation can support science to work in a more integrated way to deliver these solutions, thus utilising multidisciplinary project teams to work to a common goal (i.e. interdisciplinary research).^46^ This, in itself, has potential to overcome issues of silo thinking, which can be a barrier to progress.^47^ As described by Toomey *et al*.:^48^ interdisciplinary research ‘is most often connected with applied research that starts with a real‐world question and uses different disciplinary ideas and … can result in novel, unexpected answers to familiar, timeworn questions’. The complexity of policy challenges (in our case, balancing food production and supply with environmental goals) and data governance requires inter‐ and transdisciplinary approaches.^49^

#### *Organisational change*

Digital technology is developing more quickly than the scientific validation, compromising science's ability to advise industry on the validity or usefulness of these technologies in the value chain. There is a risk that if agricultural research organisations do not adapt they become irrelevant, overtaken by new scientific methods and partnerships between innovative clusters of researchers and business. Brynjolfsson & McAfee^7^ nicely articulate the need for organisational change to capitalise on the new opportunities arising from computing power and analytics: ‘organisational innovation: co‐inventing new organizational structures, processes and business models that leverage ever‐advancing technology and human skills’. We suggest that priorities for organisational change (Table 2) include reassessment of business models, encouraging innovation, new partnerships, and upskilling project management.

##### *New business models of research*

The transition science has to make from traditional research, based on field experiments or limited observations from practice (small data), to the use of big data from practice does not only bring technical challenges. Much as we have discussed issues of data ownership and trust and how these affect stakeholders along the value chain, these same issues are pertinent to scientists trying to leverage value from data. The scientific value of big data will often derive from combining data from multiple sources; the current research business models for accessing and using such data may need to change to enable transparent data sharing. The delivery of research‐derived solutions may also evolve: outputs from digitalised‐based science may not necessarily be scientific papers – still the mainstay of peer review. The increasing democratisation of AI (and in some cases data) means that systems can be developed without statistical validation or science involvement. Innovation then is no longer necessarily only the domain of researchers in research organisations; democratisation of innovation.^50^ The funding and project management required to optimise outcomes from digital‐based programmes has the potential to be different to conventional funding methodologies based on hypothesis‐driven reductionist science carried out in commercial or public‐funded research organisations. These indicate that the business models for science, and science funding, are likely to evolve with increasing digitalisation.^51^

##### *Innovation*

However, perhaps the greatest organisational change will be the requirement to think about and plan for scenarios well beyond business as usual. Kelly *et al*.^41^ reported that many organisations cannot visualise the paradigm shift that digital agriculture could cause, focusing more on the here and now; that is, at the tactical and operational levels. They argue that, with many organisations, it is not seen as game changing or disruptive but is considered another tool for continuous improvement and incremental gains. This is as equally applicable to researchers and research institutes, and it is beholden on researchers to think beyond this horizon if the desired benefits of digital agriculture are to be realised.

##### *Upskilling*

Although these institutional challenges will need to be addressed, researchers will also need to embrace use of digital technologies; they will need to have the skills and technological capability to operate in this field; and they will need to be able to apply scientific knowledge to capture, interpret, share, and apply digital information appropriately. One way for research organisations to develop new skills and capabilities is through partnerships or collaborations among partners that have not traditionally worked together; for example, linking agricultural scientists with technology companies (‘technology ecosystems’).

The predicted pace of technology progress^17^ will also provide challenges for the traditional 3–5 year funding cycles, the annual funding calls, and the time that it traditionally takes to achieve scientific progress.^52^ We cannot completely mitigate against these risks, but they can be minimised by building research programmes that are agile and responsive in their project management, as well as working with funders to allow these attributes to be built into project proposals. Thus, upskilling also extends to the way we design and manage our projects.

Perhaps the biggest challenge to agriculture‐based science and scientists, however, is that digitalisation of research and innovation will challenge our identity,^53^ and hence the need to change the way we work to meet this challenge, and opportunity.

## CONCLUSIONS

Digital technologies and their interconnectedness through the Internet of Things offer potential for solutions to produce more food more sustainably and link consumers and farmers. However, it is clear that embedding digital agriculture, and equitably sharing the potential benefits it has to offer, will require significant agricultural system changes and the need to address critical socio‐ethical, as well as technical issues. Science and scientists will play a critical role in navigating this change. Digitilisation of science offers both a challenge and an opportunity to science playing this role.
